# Reservation in postgraduate education for government in-service doctors: a case study from Andhra Pradesh

**DOI:** 10.1186/1753-6561-6-S1-P6

**Published:** 2012-01-16

**Authors:** Zubin Shroff, Seema Murthy, Krishna Rao

**Affiliations:** 1 Public Health Foundation of India, New Delhi, India; 2 Indian Institute of Public Health, Hyderabad, India

## Introduction

In order to achieve health for all, it is essential to attract and retain health workers in rural India, a need recognised by the National Rural Health Mission (NRHM), a flagship programme of government of India.

Since the majority of allopathic doctors aspire to do specialisation, making it easier for doctors serving in rural areas to get admission to specialist medical postgraduate courses is a logical step towards attracting fresh medical graduates to serve in rural health services.

In this study, we explore one such strategy used by the state of Andhra Pradesh. The state has reserved 50% of seats in postgraduate pre- and para-clinical disciplines, and 30% of seats in postgraduate clinical disciplines for candidates who have served in government health services. Students using this scheme have to sign a bond of INR 20,00,000 (USD 42946.1) and are required to serve in state government health services for the period of five years after completing their postgraduate education. If they leave the government health services before a minimum of five years period, they are obliged to pay the bond amount and refund the salary amount received after their postgraduate course. Doctors having served for two years in tribal areas, or three years in rural areas, or five years in urban areas become eligible for these reserved seats.

## Methods

We used a qualitative case study methodology in which we interviewed a large number of stakeholders including government officials, health system managers, and serving medical officers. This was supplemented with quantitative data on the scheme obtained from departments of health, medical and family welfare in Andhra Pradesh.

## Results

Our study findings suggest that admission to postgraduate education is a powerful incentive for attracting fresh medical graduates to rural areas. Use of reservation for the in-service doctors increases the probability of getting admission to postgraduate courses. Over the time the number of people using this scheme has increased, possibly due to increasing difficulty in getting admission to postgraduate courses otherwise.

There is a mismatch between the specialist disciplines available through the postgraduate in-service quota and the need for specialists in these disciplines in government health services. There were also concerns expressed about the academic performance of in-service candidates. Finally there was little information on the enforcement of the bond.

## Discussion

The strategy of reserving seats in postgraduate medical courses is successful if the filling of vacancies (for doctors) in government health services is the criteria. However, it is pertinent to ask whether attracting medical graduates whose sole interest, in many cases, is getting an admission to postgraduate courses is a good strategy to provide quality healthcare to people. It is also important to encourage academic interest in rural health problems among doctors during their rural health services.

Finally, there is a need to rationalise the scheme and align the supply of in-service postgraduate specialists with demand for specialists in these disciplines in Andhra Pradesh. The directorate of medical education has noticed this issue and made recommendations in this regard. Finally, there is need to maintain information on in-service postgraduate candidates that would enable enforcement of compliance with the bond.

